# An Infant Formula with Large, Milk Phospholipid-Coated Lipid Droplets Supports Adequate Growth and Is Well-Tolerated in Healthy, Term Asian Infants: A Randomized, Controlled Double-Blind Clinical Trial

**DOI:** 10.3390/nu14030634

**Published:** 2022-02-01

**Authors:** Oon Hoe Teoh, Tan Pih Lin, Marieke Abrahamse-Berkeveld, Antoinette Winokan, Yap Seng Chong, Fabian Yap, Maya Marintcheva-Petrova, Eline M. van der Beek, Lynette P. Shek

**Affiliations:** 1 Department of Paediatrics, KK Women’s and Children’s Hospital, Singapore 229899, Singapore; teoh.oon.hoe@singhealth.com.sg (O.H.T.); tan.pih.lin@singhealth.com.sg (T.P.L.); fabian.yap.k.p@singhealth.com.sg (F.Y.); 2 Danone Nutricia Research, 3584 CT Utrecht, The Netherlands; maya.petrova@danone.com; 3 Nutricia Research, Singapore 138671, Singapore; antoinette.winokan@danone.com; 4 Singapore Institute for Clinical Sciences, Agency for Science, Technology and Research, Singapore 119228, Singapore; obgcys@nus.edu.sg (Y.S.C.); paeshekl@nus.edu.sg (L.P.S.); 5 Department of Obstetrics and Gynaecology, Yoo Loo Lin School of Medicine, The National University of Singapore and National University Health System, Singapore 119228, Singapore; 6 Department of Paediatrics, University Medical Centre Groningen, University of Groningen, 9700 RB Groningen, The Netherlands; e.m.van.der.beek@umcg.nl; 7 Department of Pediatrics, Yoo Loo Lin School of Medicine, The National University of Signapore and National University Health System, Singapore 119228, Singapore

**Keywords:** lipid droplet structure, safety, growth adequacy, infant formula

## Abstract

Lipids are essential for healthy infant growth and development. The structural complexity of lipids in human milk is not present in infant milk formula (IF). A concept IF was developed mimicking more closely the structure and composition of human milk fat globules. The current study evaluates whether a concept IF with large, milk phospholipid-coated lipid droplets (mode diameter 3 to 5 μm) is equivalent to standard IF with regard to growth adequacy and safety in healthy, term Asian infants. In this randomized, double-blind, controlled trial, infants were randomized after parents decided to introduce formula. Infants received a standard IF with (Control) or without the specific prebiotic mixture scGOS/lcFOS (9:1 ratio; Control w/o prebiotics), or a Concept IF with large, milk phospholipid-coated lipid droplets and the prebiotic mixture. A group of 67 breastfed infants served as a reference. As a priori defined, only those infants who were fully intervention formula-fed ≤28 days of age were included in the equivalence analysis (Control n = 29; Control w/o prebiotics n = 28; Concept n = 35, per-protocol population). Primary outcome was daily weight gain during the first four months of life, with the difference between the Concept and Control as the key comparison of interest. Additionally, adverse events, growth and tolerance parameters were evaluated. Equivalence of daily weight gain was demonstrated between the Concept and Control group after additional correction for ethnicity and birthweight (difference in estimated means of 0.1 g/d, 90%CI [−2.30, 2.47]; equivalence margin +/− 3 g/d). No clinically relevant group differences were observed in secondary growth outcomes, tolerance outcomes or number, severity or relatedness of adverse events. This study corroborates that an infant formula with large, milk phospholipid-coated lipid droplets supports adequate growth and is well tolerated and safe for use in healthy infants.

## 1. Introduction

Human milk provides a complete supply of nutrients to optimally support infant growth and development in early life [1]. Exclusive breastfeeding for the first 6 months and in combination with complementary foods up to 2 years and beyond is the universally recommended feeding mode for infants by the World Health Organization [2]. In case breastfeeding is not possible, the provided infant formula (IF) must be safe and suitable to meet the nutritional requirements of infants, promoting their growth and development [3]. 

Lipids in human milk provide almost half of the caloric intake of infants [4]. The total lipid content is relatively stable and crucial in fulfilling nutritional needs, although the lipid composition in human milk changes over the course of lactation [5,6]. Lipids in human milk are present as lipid droplets with a volume-based mode diameter of 3–5 μm and are enveloped by a tri-layered membrane mainly consisting of phospholipids, membrane-specific proteins and cholesterol [7,8,9]. The structural complexity of these lipid droplets originates from their synthesis and excretion by the epithelial cells of the mammary gland. Lipids in IF, in contrast, are present as lipid droplets with a volume-based mode diameter of ~0.5 µm with mainly proteins adhered to their outer surface area [9].

Given the lasting impact of lipids in early life on infants’ development [10,11], the lipid quality of IFs has received particular interest over the past few decades [12]. Adaptations in production processes have resulted in the development of a concept IF containing larger lipid droplets (3–5 μm) with a thin interface of milk phospholipids, other polar lipids, (glyco)proteins and cholesterol, mimicking some of the structural aspects of lipids in human milk [13]. In line with previous findings on the physiological properties of lipid droplet characteristics [14,15,16], this concept IF was found to alter in vitro lipid digestion kinetics [17] and postprandial lipid responses in adult men [18]. In rodent models of nutritional programming, it prevented excessive fat accumulation and adverse metabolic outcomes [19,20,21], as well as improved specific cognitive behaviors [22], compared to a standard IF. Hence, it was postulated that introducing large, phospholipid-coated lipid droplets may bring the physiological properties of IF, i.e., digestion kinetics and postprandial absorption, closer to those of human milk, potentially resulting in persistent nutritional (metabolic) programming effects [12,13,23,24] 

In a randomized, double-blind, controlled trial in Singapore, the impact of a concept IF with large, milk phospholipid-coated lipid droplets comprising only vegetable lipids on infant growth and development as well as long-term childhood health outcomes was evaluated [25]. One of the predefined objectives was a stringent evaluation of the safety and nutritional adequacy of this concept IF. In compliance with current guidance for the evaluation of the safety and suitability of IFs, this study investigates growth adequacy, in Asian infants consuming this concept IF as the sole source of nutrition during their first four months of life (daily weight gain as primary outcome), as well as its impact on gastrointestinal tolerance and adverse events. 

## 2. Materials and Methods

### 2.1. Participating Centers

This study was conducted at the National University Hospital and the KK Women’s and Children’s Hospital in Singapore, in compliance with the principles of the World Medical Association Declaration of Helsinki (59th WMA General Assembly, Seoul, October 2008), International Conference on Harmonisation (ICH) guidelines for Good Clinical Practice (GCP, September 1997), as well as Singapore’s regulatory requirements. This study and the associated follow-up were approved by the Domain-Specific Review Board for the National University Hospital (approval no. 2011/01838) and SingHealth Institutional Review Board for KK Women’s and Children’s Hospital (approval no. 2011/635/E). The study was registered at a public register with identifier numbers NCT01609634 and NCT02594683 (www.clinicaltrials.gov). Written informed consent was obtained from all parents/guardians before enrolment.

### 2.2. Study Design

Details of the overall design of the trial were reported previously [22]. Briefly, a randomized, double-blind, controlled, 3-arm, parallel-group intervention trial was performed with three IFs that differed in lipid droplet structure and/or presence of prebiotics to assess the impact on infant growth and development and allergy outcomes at up to 5 years of age. Infants were enrolled in the study in the first month of life and block-randomized (block size of six) on a 1:1:1 basis, stratified by sex and age at time of randomization (age ≤ 28 days, 29 days ≤ age ≥ 17 weeks or age > 17 weeks) to receive one of three formulas until 12 months of age. Formulas were coded by the sponsor using the letter codes A, B, C, D, E and F. A study-independent statistician created the randomization number lists using the PLAN procedure of SAS statistical software (SAS Institute Inc., Cary, North Carolina, USA), with each number assigned to a letter code. Upon enrolment, the electronic case report form (eCRF) provided the randomization code after the respective eCRF information was completed. Both the investigators and the infants’ parents were blinded to the formulas. The timing of randomization was flexible and occurred at any moment during the first year of life, fully depending on the moment parents decided to start formula feeding (either with or without continued breastfeeding). This design was chosen since, in compliance with local ethical standards, it respects and is unlikely to interfere with breastfeeding practices. As such, it is anticipated to reflect the real-life milk feeding practices. Until 17 weeks of age, apart from formula or breastmilk, only use of water, tea or rehydration solutions, drops or syrups (vitamins, minerals, medicines) was allowed. 

Respecting the guidelines for stringent safety evaluation [3,26], we only considered infants that were fully study formula-fed by 28 days of age as the primary analysis population for the growth adequacy evaluation up to 17 weeks. Analysis of safety and tolerance outcomes included all infants consuming study formula before 17 weeks of age, irrespective of the age at formula introduction or amount consumed. Additionally, infants that were fully breastfed for the first four months of life served as a reference group.

### 2.3. Subjects

Healthy term infants of Chinese, Malay or Indian ethnicity, with a gestational age between 37 and 42 weeks, postnatal age ≤ 28 days and a birthweight and head circumference between the 3rd and 90th percentile according to the Fenton growth chart [27], were eligible for participation. Exclusion criteria were defined as illnesses that could interfere with the study, special dietary needs, diagnosed maternal hepatitis B or HIV, maternal or infant participation in any other study or investigator’s uncertainty about the ability of the parents to comply with the protocol requirements. Initially, to minimize the ethnicity impact on infant growth outcomes, the study aimed to include only infants of Chinese ethnicity. However, inclusion criteria were adapted following slow recruitment rates in the first 6 months. As such, (1) infants of the two other main ethnic groups in Singapore, Malay and Indian, could be enrolled as well; (2) the lower threshold for birthweight changed to the 3rd percentile according to the Fenton growth chart, so as to include infants who were constitutionally small in this Asian population with lower birthweight values. In addition, a head circumference criterion was added as assessment and confirmation of infants’ health at inclusion.

### 2.4. Study Products

The intervention formulas were iso-caloric (66 kcal/100 mL), containing similar amounts of protein (1.3 g/100 mL) and lipids (3.4 g/100 mL), and were manufactured per good manufacturing practices (ISO 22000) and compliant with Directive 2006/141/EC (Table 1). All intervention formulas had a whey:casein ratio of 60:40 and had a similar lipid content and fatty acid composition, mainly comprising vegetable oils, with a DHA to ARA ratio of 1:2. The differences between the three study IFs were (1) the size of their lipid droplets and coating of their lipid droplets and/or (2) the presence of the specific prebiotic mixture short-chain galacto-oligosaccharides and long-chain fructo-oligosaccharides (scGOS/lcFOS; 9:1). Two of the intervention formulas were standard IFs containing lipid droplets with a volume-based mode diameter of 0.5 μm and proteins as main emulsifiers. The sole difference between these two formulas was the absence (Control w/o prebiotics) or presence (Control) of 0.8 g/100 mL of the specific scGOS/lcFOS (9:1) mixture. The third intervention formula, the Concept IF, contained the aforementioned prebiotic mixture and had lipid droplets with a volume-based mode diameter of 3 to 5 μm and an interface predominantly composed of added milk phospholipids following an adapted production process [13]. 

### 2.5. Measurements

The primary outcome measure of the current study was the daily weight gain (g/d) from randomization (formula group) or enrolment (breastfed reference) until 17 weeks of age. Additional outcome parameters included length, head circumference, tolerance parameters, adverse events and medication use.

After enrolment in the study, visits took place at 4, 8, 13 and 17 weeks of age. Infants had an additional randomization visit whenever parents decided to introduce formula. Subject characteristics were collected at the screening visit and the parental characteristics and demographics were collected during the visit at 4 weeks of age. Anthropometric parameters were measured at enrolment and each visit thereafter. Infants were weighed naked to the nearest gram on a calibrated electronic weighing scale. Recumbent length of the infants was measured to the nearest 0.1 cm with a length board. A non-stretchable measuring tape was used to measure head circumference. Anthropometric measurements were taken twice, with a third taken if a substantial difference between measurements was recorded (>50 g for weight and >5 mm for length and head circumference). The measurements were averaged for the statistical analysis. 

Daily study product intake, gastrointestinal symptoms and stool characteristics were recorded by the parents in diaries spanning the 7-day period preceding each visit. Severity of gastrointestinal symptoms (vomiting, regurgitation, cramps and diaper rash), if any, was recorded once a day on a 4-point scale (absent, mild, moderate and severe). Stool consistency was scored for each stool passed, on a 4-point adjectival scale using pictures (watery, soft, formed or hard stools with values from 1 to 4, respectively), according to the “Amsterdam” stool form scale [28]. During the study, every (serious) adverse event was documented by the investigators at each visit, including the nature, onset, duration, severity and seriousness, (possible) relationship with the study product use, any actions that were taken and the outcomes. All (S)AEs were followed up by the investigator until they had abated or until a stable situation had been reached. 

### 2.6. Statistics

The primary outcome of the study was the daily weight gain (g/d) from randomization (or enrolment for breastfed infants) until 17 weeks of age. The primary comparison of interest was between the Concept formula and the Control formula group, both containing prebiotics. Additionally, the comparison of both these groups versus the Control w/o prebiotics, originally included in the study to evaluate impact of prebiotics on (long-term) allergy outcomes, was provided. 

Equivalence in the fully formula-fed per-protocol population was to be demonstrated when the two-sided 90% confidence interval (CI) of the difference in means of daily weight gain lay within the pre-defined equivalence margins of ±3 g/d [29]. The required sample size for two one-sided statistical testing using a SD of 6.0 g/d, α = 0.05, a power = 0.80 and assuming no daily weight gain differences between two parallel groups was 70 infants per intervention group. Assuming a drop-out rate of 15%, a total of 249 infants that were fully formula-fed ≤28 days of age (83 per intervention group) had to be enrolled. Based on the assumption that 27% of the infants in this population would be fully formula-fed by the age of 28 days, a total sample size of 922 enrolled subjects (in the intervention groups) was required [25]. 

Several analysis populations were used for the evaluation of the study outcomes. In the all-subjects-treated (AST) analysis, the data of all infants who started consumption of the study product ≤4 months of age were used, whereas, for the intention-to-treat (ITT) analysis, the data of all infants randomized to study product ≤4 months of age were used. Infants fully breastfed until 4 months of age served as a reference. In the per-protocol (PP) population, eligibility of data was assessed on visit level and growth data of breastfed and formula-fed infants were excluded when other infant formulas or solid foods were consumed for >3 days before any visit until 17 weeks of age. For drop-outs, the available data until drop-out were considered in the statistical analyses; no data imputation was performed. 

Equivalence analyses for weight gain, length gain and head circumference gain were performed in the fully formula-fed PP population using a longitudinal mixed-effects model that described the development of growth as a quadratic function of time (infant’s age) and also accounted for the flexible start of study product intake (within first 4 months of age) via a quadratic function of time since start of study product intake. The basic model included only the stratification factors as a fixed effect and the interaction with time and time squared, whereas an extended model also included infant birthweight and ethnicity (Chinese/non-Chinese), and the interaction of ethnicity with time and time squared, given their imbalances between intervention groups. Random effects for subject and its interaction with time and time squared with unstructured covariance structure were used. As a sensitivity equivalence analysis, the basic model was to be fitted on the fully formula-fed ITT population. Anthropometric data were converted to z-scores using the WHO growth standards [30]. 

Tolerance outcome evaluation was based on the AST population, comprising infants who had taken at least one feeding of intervention product, and only included subjects’ diary data when at least 3 informative days were available (per visit period). The timing of randomization was flexible and, to evaluate the intervention products most stringently, the diary data of randomized infants before their first formula consumption (when still being breastfed) were not considered in the statistical analysis comparing intervention groups. These data were allocated to the group of breastfed infants instead. Median stool consistency score was calculated for each subject at each visit and is further referenced as the stool consistency score. The percentage of days with at least one watery stool or with at least one hard stool or no stool was calculated. The incidence of diarrhea and constipation was derived from the stool frequency and consistency score based on the individual diary information of the period prior to a visit. Derived from the WHO definition, diarrhea was defined as present when a subject passed “3 or more watery stools on at least one day”. Constipation was defined as present if a subject passed “at most 2 stools per week, all of hard consistency” (adapted Rome III criteria [31]). For the evaluation of other GI symptoms, i.e., cramps, diaper rashes, regurgitation and vomiting, the severity per subject and visit was based on the worst severity reported (categories absent, mild, moderate, severe) over a 7-day period prior to each visit. 

For the safety evaluation, the AST population was used based on infants who had taken at least one feeding of an intervention product. Pre-specified groups of adverse events were created based on their clinical relevance: skin allergies, respiratory allergies, any allergies, upper respiratory tract infections, lower respiratory tract infections, other respiratory tract infections, gastrointestinal infections, other infections and gastrointestinal symptoms.

To compare the groups with respect to average daily stool frequency, stool consistency score and the percentages of days with at least one watery or hard (no) stool, the Mann–Whitney U test (MW) test was used, and for diarrhea, constipation and adverse events, the Miettinen and Nurminen score (MN) was used. Categorical response parameters with more than two categories (cramps, diaper rashes, regurgitation, vomiting) were analyzed by using Chi-square tests (C; Fisher’s exact tests in case sparse cells occurred; FE). All statistical analyses were performed using SAS^®^ (SAS Enterprise Guide 4.3 or higher) for Windows (SAS Institute Inc., Cary, NC, USA). 

## 3. Results

### 3.1. Study and Subject Characteristics

An independent data monitoring committee conducted an interim analysis to perform safety surveillance, which had been ongoing for one year, and the committee provided the advice to continue the study without modifications. However, due to slow recruitment rates and a substantially lower than expected number of fully formula-fed infants in the first month of their life, as well as unforeseen raw material sourcing issues that could have potentially undermined Concept IF availability, the recruitment of infants was stopped prematurely [25]. Between July 2012 and August 2014, a total of 590 subjects were screened for eligibility and 539 subjects were enrolled in the study. No apparent differences were observed between subject and demographic characteristics between the three intervention groups in the ITT population (Supplementary Table S1).

**Figure 1:**
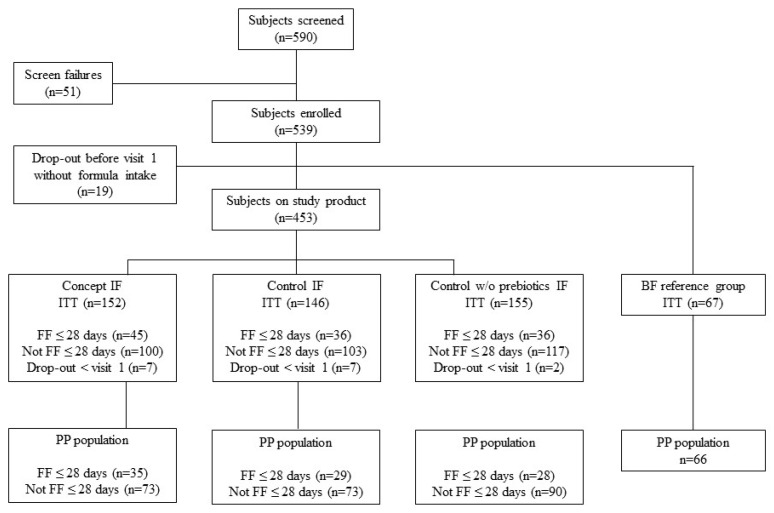
Flow chart of progression of infants during the study. ITT, intention-to-treat population; FF, fully formula-fed; PP, per-protocol population. Numbers of the PP population indicate group sizes at 1 month of age; protocol compliance was based on visit level and led to partial exclusion of 16 subjects up to 17 weeks of age (Concept: 3 subjects, Control: 8 subjects, Control w/o prebiotics: 5 subjects).

Only 117 subjects were fully formula-fed by 28 days of age, which is a lower number (due to the premature stop of recruitment) than the original estimated required sample size of 249 infants for the equivalence analysis. A total of 67 infants were included in the breastfed reference group (Figure 1). Twenty-two infants that were fully formula-fed from ≤28 days of age dropped out before 17 weeks of age (19%), with 16 subjects even before 4 weeks of age. The reasons for early termination were the withdrawal of informed consent (Concept: five subjects, Control: six subjects), withdrawal after serious adverse events (Control w/o prebiotics: one subject) or use of non-study formula or introduction of complementary feeding (Concept: three subjects, Control: one subject). The per-protocol population (PP) fully formula-fed at 1 month of age consisted of 92 subjects, with a total of 35, 29 and 28 subjects in the Concept, Control and Control w/o prebiotics groups, respectively. For the PP population, protocol compliance was based on visit level and led to the partial exclusion of 16 subjects up to 17 weeks of age (Concept: three subjects, Control: eight subjects, Control w/o prebiotics: five subjects). All infants in the breastfed reference group completed the visits up to 17 weeks of age, with only one infant being non-compliant to the protocol (use of non-study formula). The demographic data indicated that there were apparent differences between the formula groups for the infants of the PP population who were fully formula-fed ≤28 days of age (Table 2); a higher prevalence of boys, infants of Chinese ethnicity and the lowest mean birthweight was observed in the Concept group versus both Control groups. In the Control group, the prevalence of C-section birth was half versus the two other intervention groups. In the breastfed group, our reference group, the prevalence of Chinese ethnicity and level of maternal education was highest and prevalence of maternal smoking and overweight/obesity lowest. 

### 3.2. Study Product Intake

No apparent differences in mean daily formula intake (ml/day) or intake per kg body (mL/kg/day) were observed across intervention groups (Supplementary Table S2). 

### 3.3. Growth Outcomes

Equivalence in daily weight gain was not demonstrated for any of the comparisons between formula intervention groups in the PP population when using the basic model (data not shown). Equivalence in daily weight gain (g/d) from randomization to 17 weeks of age was demonstrated for the Concept vs. Control formula group (mean (SE) of 30.05 (0.95) g/d vs. 29.96 (1.12) g/d; primary comparison) in the PP population after adjusting for birthweight and ethnicity in the model (difference in estimated means of 0.09 g/d, 90%CI [−2.30, 2.47]; extended model with intervention groups only). Equivalence in daily weight gain was not demonstrated comparing either the Concept formula or the Control formula group versus the Control w/o prebiotics group (mean (SE) of 30.84 (1.13) g/d; extended model with intervention groups only). Compared to the breastfed reference group (mean (SE) daily weight gain of 30.52 (0.77) g/d), equivalence in daily weight gain was demonstrated for the Concept and Control formula groups (difference in estimated means of 0.31 g/d, 90% CI [−1.76, 2.39 g/d] and 0.54 g/d 90% CI [−1.81, 2.89 g/d], respectively; extended model with intervention groups and breastfed reference group), but not for the Control w/o prebiotics group (difference in estimated means of 1.06 g/d, 90%CI [−1.33, 3.46 g/d]). In the ITT population, equivalence in daily weight gain was confirmed for the Concept vs. Control group (difference in estimated means of 1.01 g/d, 90%CI [−0.94, 2.97 g/d]) as well as the Concept vs. Control w/o prebiotics group (difference in estimated means of −0.75 g/d, 90%CI [−2.69, 1.19]), but not for the Control vs. Control w/o prebiotics group (difference in estimated means of −1.76 g/d, 90%CI [−3.79, 0.26]; extended model including intervention groups only). Mean weight, length and head circumference were not statistically significantly different (at 5% significance level) for any of the intervention group pairwise comparisons at any visit until 17 weeks of age for the PP and ITT populations (extended model including intervention groups and breastfed reference group; data not shown). 

**Figure 2:**
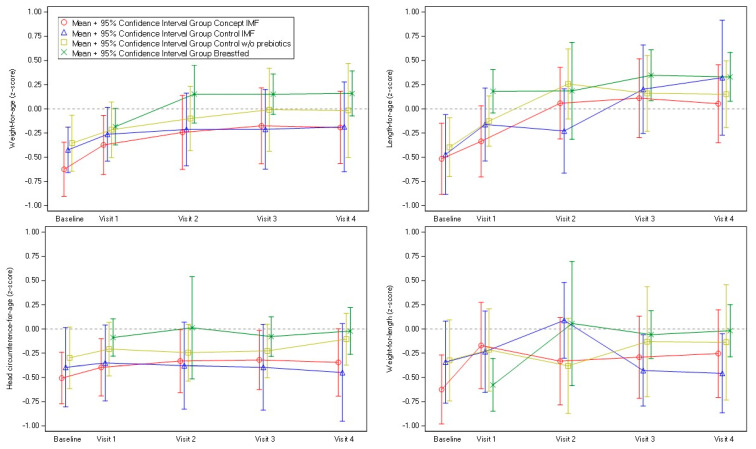
Mean (±95%CI) weight-for-age, length-for-age (left and right upper panel), head circumference-for-age and weight-for-length (left and right lower panel) WHO growth standard z-scores for the Control w/o prebiotics IF, Control IF, Concept IF and the breastfed reference group of the PP population. IF, infant milk formula. Baseline values of intervention groups collected at randomization visit prior to visit 1.

The mean weight-for-age, length-for-age and head circumference-for-age WHO z-score values were below zero (close to −0.5 z-score) at enrolment in all formula groups of the PP population (Figure 2). During the intervention period, these mean z-score values remained stable (head circumference-for-age) or slightly increased over time (weight-for-age and length-for-age), with values within the ±0.5 z-score bandwidth, indicative of adequate growth. As a reference, the infants of the breastfed reference group in the PP population had the highest mean z-score values at baseline, a pattern that persisted throughout the intervention period, indicated by the consistent numerically higher mean z-scores. Similar findings were observed for the ITT population (data not shown).

### 3.4. Gastrointestinal Tolerance

The analysis of tolerance outcomes was performed using the parent-reported information up to 17 weeks of age of the total AST population (Figure 1; 152 subjects in Concept group, 146 subjects in Control group and 155 subjects in Control w/o prebiotics group). No significant differences in daily stool frequency were observed between the intervention formula groups until 17 weeks of age, apart from a slightly lower daily stool frequency in the Control w/o prebiotics group versus the other two intervention groups at 17 weeks of age (Table 3; *p* = 0.026 vs. Test and *p* = 0.023 vs. Control; MW). Compared to formula-fed infants, the breastfed reference group consistently showed a higher daily stool frequency ranging from 4.9 to 1.3 times per day (median breastfed infants) versus 2.6 to 0.9 (medians across intervention groups) from 1 to 4 months of age, respectively (Table 3). 

At all timepoints during the study, the median of the stool consistency score was “soft” (value of 2) in all groups (data not shown). However, infants fed the Concept IF had a statistically significant different stool consistency score compared to both Control groups, with a lower mean consistency score reported for the Concept IF at 1 and 4 months of age and compared to the Control w/o prebiotics group at 3 months of age as well (Table 3). The mean stool consistency scores between the Concept IF and breastfed reference group were not statistically significantly different at any timepoint (Table 3). Evaluating the percentage of days that a subject had at least one watery stool, higher percentages were observed for the Concept IF group compared to the Control w/o prebiotics (at 1, 3 and 4 months of age) and the Control group (at 4 months of age only). No statistically significant differences were found between the Concept and the breastfed reference group at all timepoints. No clinically relevant differences were observed in the percentages of subjects with diarrhea (based on diary data) between formula intervention groups, nor compared to the breastfed group (Supplementary Table S3). In the formula intervention groups, only a few isolated cases of constipation were observed between 2 and 4 months of age, without clinically relevant differences between any of the groups, in line with the similar percentage of days with at least one hard stool or no stool reported for the intervention groups during these four months (Table 3). During the study period, most infants across the intervention and breastfed reference groups did not experience any cramps (ranging from 84.4% to 98.5% across visits and groups; data not shown). No statistically significant differences in the distribution of percentages of subjects with no, mild, moderate or severe cramps were observed between randomized groups, with values close to the breastfed reference group. No statistically significant differences in the percentage of subjects with absent, mild, moderate or severe diaper rash were observed, apart from a lower prevalence in the Control w/o prebiotics group (14.7%) versus all other groups at 1 month of age (ranging from 27.8% to 30.7%; *p* < 0.05; Chi-Square/FE). Some sporadic severe cases of diaper rash were observed in both control groups, but not in the Concept group or in the breastfed reference group. The percentages of infants with absent, mild, moderate or severe regurgitation were not statistically significantly different between intervention groups, and regurgitation declined over time, apart from the distribution across regurgitation categories for Concept IF versus Control w/o prebiotics IF group at 2 months of age (Supplementary Table S3). As a reference, the breastfed group had consistently higher percentages of infants with regurgitation during the first 4 months compared to all intervention groups, but this difference in distribution across regurgitation categories was only statistically significant at 1 month of age (*p* < 0.02; Chi-square/FE). No statistically significant differences in the distribution of vomiting categories were observed between intervention groups, with values close to the breastfed reference group. In the control groups, some isolated cases of vomiting were apparent, with three severe cases reported at month 3 for the Control w/o prebiotics group, whereas three severe cases were observed at 4 months of age in the Concept group. 

In a sensitivity analysis in the subgroup of infants in the AST population that were fully formula-fed before 28 days of age only (AST-FF), similar findings in tolerance outcomes were observed.

### 3.5. Adverse Events

The evaluation of adverse event outcomes was performed using the collected information up to 17 weeks of age for the full AST population. Additionally, as sensitivity analysis, the subgroup of infants in the AST population that were fully formula-fed before 28 days was evaluated (AST-FF, n = 117). 

Overall, 71 serious adverse events (SAE) were reported for 60 subjects in the intervention groups (13% of total number of subjects) from enrolment until 17 weeks of age for the AST population. In the AST-FF subgroup, 28 SAEs were reported for 21 subjects in the intervention groups (18% of total number of subjects). For comparison, in the breastfed reference group, five SAEs were reported in four infants (6.0%) during the same period. The percentage of subjects with one or more SAEs overall, as well as their severity, which was mostly moderate to severe, was neither statistically significant different between formula groups in the AST population nor in the AST-FF subgroup. Most of the reported SAEs were related to infections and infestations, with 21 events in 18 subjects (11.8%), 17 events in 14 subjects (9.6%) and 13 events in 13 subjects (8.4%) for the Concept, Control and Control w/o prebiotics group of the AST population, respectively. None of the SAEs in any group was considered to be related to the intake of the study product, except for a severe case of regurgitation in the Control w/o prebiotics group, which was assessed as possibly related. 

A total of 790 AEs was reported in 334 subjects of the intervention groups (74%) for the AST population, with 234 AEs reported in 92 subjects (79%) of the AST-FF subgroup. No statistically significant differences in the percentages of subjects with AEs were observed between intervention groups in the full AST population. A higher frequency of AEs was observed for the Concept IF in the AST-FF subgroup versus both the Control and Control w/o prebiotics groups (*p* < 0.05 MN; 114 events in 41 subjects (91%), 64 events in 26 subjects (72%) and 56 events in 25 subjects (69%), respectively). The most frequently reported adverse events were (related to) upper respiratory tract infections, gastrointestinal function or allergies. However, differences observed in the type of AEs (preferred terms) between formula intervention groups in the AST population and AST-FF subgroup were not statistically significant nor clinically relevant. None of the adverse events that were documented during the study were considered related to the study product by the investigators. Based on the outcomes described above, there was no safety concern related to the occurrence of any (S)AEs during the study. 

## 4. Discussion

This double-blind, randomized, controlled trial evaluated the safety and nutritional adequacy of a concept infant milk formula with lipid droplets that were large and coated with milk phospholipids, features that were inspired by the large-sized and tri-layered membrane-enveloped fat globules in human milk [13]. After adjustment for confounding factors, we demonstrated an equivalent daily weight gain for infants fed the Concept IF compared to those fed a Control IF up to 17 weeks of age. All intervention formulas supported adequate growth, were well tolerated and no major safety concerns were revealed based on the number, severity, relatedness or type of (serious) adverse events observed during the first four months within this study. 

Previous studies have shown that the mere enrichment of standard formula with milk phospholipid fractions was well tolerated and supported adequate infant growth [32,33]. Recently, an infant formula with large, milk phospholipid-coated lipid droplets containing dairy lipids (48% of total lipids) was demonstrated to be safe, well tolerated and supporting adequate growth in healthy term European infants [34]. In the current study, despite the modest sample sizes of the PP population in the intervention groups, the Concept IF group demonstrated an equivalent daily weight gain compared to the Control IF group after adjustment for birthweight and ethnicity as confounding factors. Moreover, the mean infant growth outcomes in all randomized formula groups of the current study were close to the median of the WHO growth standards (within ±0.5 z-score bandwidth), indicative of adequate infant growth [35,36]. Together, these findings support the conclusion of the previous (fully powered) equivalence study [34] demonstrating that an infant formula with large, milk phospholipid-coated lipid droplets (containing dairy lipids) supports adequate infant growth. 

The WHO growth standards are based on the growth of exclusively breastfed infants from six different countries across the globe and recommended as the global standard to assess infant growth adequacy. In the current study, the infants in the randomized formula groups had mean weight-for-age, length-for-age and head-circumference-for age z-score values lower than the WHO growth standard median at birth but increasing over time to values close to the median from 2 months of age onwards. A similar pattern was observed in the infants of the breastfed reference group. Although this growth pattern deviates from those described by the WHO standards, these observations are in line with previous findings in Asian populations in which lower or steadily increasing z-score values were reported for Indian and Malay or Chinese reference cohorts, respectively [37,38,39]. Additionally, it underlines the importance of including a group of breastfed reference infants to provide a population-specific context for the interpretation of study outcomes.

As expected, the stool consistency of most formula-fed infants in the current study was categorized as “soft” stool. The prebiotic mixture scGOS/lcFOS (9:1) present in the Concept and Control IF was previously shown to stimulate intestinal colonization with bifidobacteria, resulting in beneficial effects on immune function and a stool-softening effect [40,41,42,43]. However, no apparent differences in stool characteristics were observed between the Control w/o prebiotics versus the Control IF group with prebiotics, apart from a slightly lower stool frequency and lower percentage of days with at least one watery stool in the Control w/o prebiotics group at 17 weeks. It is highly likely that the heterogeneity in milk feeding practices in the AST population, i.e., combining formula feeding with breastfeeding with large variation in exclusivity and duration, may have reduced the likelihood of observing a more substantial impact of the prebiotic mixture on stool characteristics. A slightly lower mean stool consistency score was reported at several timepoints when comparing the Concept versus both Control IF groups, possibly explained by the slightly higher occurrence of watery stools, closer to values observed in the breastfed reference group. However, given the limited scale of these differences and the proximity of the observed values in the occurrence of watery stools and diarrhea to the breastfed reference group, we conclude that the observed differences were not clinically relevant.

Overall, the reported types of (serious) adverse events are typical for young infants and no clinically relevant difference in the frequency, relatedness, type or severity of (serious) adverse events was observed between formula groups. The number and reasons for drop-out were similar between the Concept IF and Control IF groups. Moreover, none of the parameters that we assessed to evaluate tolerability indicated any adverse effects of the Concept IF. The findings of the current study support the conclusions of the aforementioned first clinical evaluation study [34] that the concept IF containing large lipid droplets is safe for use in infants. 

Finally, some limitations of this study need to be addressed. The premature stop in recruitment, in combination with the lower than expected (full) formula usage, resulted in a substantially lower sample size, with only nearly half of the originally intended population size for the growth equivalence analysis. Hence, caution should be used in the interpretation of the outcomes of the current study in relation to its nutritional interventions. The inclusion of Malay, Indian as well as Chinese infants in this study may have introduced additional confounding, given the previously observed differences in growth trajectories [33,34,35]. Moreover, the milk feeding characteristics of these ethnic populations might be different as well, e.g., evident from the higher number of Chinese infants in the breastfed reference group versus the randomized IF groups. Moreover, the limited number of infants resulted in some imbalances in demographic characteristics, such as differences in birthweight and ethnic representation in the randomized groups, which we corrected for in the equivalence analysis, which could also hamper interpretation for the general population. Lastly, it is of importance to state that we did not adjust for multiplicity in the evaluation of gastrointestinal tolerance and adverse event outcomes, so any reported differences should be interpreted with caution. However, we would like to emphasize that we have evaluated observed differences between treatment groups primarily on their potential clinical relevance rather than on statistical significance. 

In conclusion, the results of the current study suggest that a concept IF with large, milk phospholipid-coated lipid droplets supports adequate growth and is well tolerated and safe for use in infants. The potential long-term effect of lipid droplet characteristics in infant nutrition on growth trajectories, body composition and metabolic development, as well as other health outcomes, remains to be elucidated. 

## Figures and Tables

**Table 1:** Composition of the intervention products.

Per 100 mL	Control w/o Prebiotics IF ^1^	Control IF	Concept IF
Energy (kcal)	66	66	66
Fat (g)	3.4	3.4	3.4
Vegetable oil (g)	3.3	3.3	3.2
Dairy lipids (g)	-	-	0.2
Non-dairy lipids (g)	0.1	0.1	
Saturates	1.5	1.5	1.5
Monounsaturates	1.3	1.3	1.3
Polyunsaturates	0.6	0.6	0.6
Linoleic acid (mg)	448	447	436
Alpha linolenic acid (mg)	83	82	81
Arachidomic acid (mg)	11	11	11
Eicosapentaenoic acid (mg)	1.4	1.4	1.8
Docosahexaenoic acid (mg)	6.4	6.4	6.4
Milk phospholipids (mg)	-	-	54.4
Soy phospholipids (mg)	4.5	4.5	-
Protein (g)	1.3	1.3	1.3
Whey protein (g)	0.8	0.8	0.8
Casein (g)	0.5	0.5	0.5
Carbohydrates (g)	7.6	7.3	7.3
scGOS/lcFOS (9:1) (g) ^2^	-	0.8	0.8
Vitamins			
Vitamin A (μg RE)	50	50	50
Vitamin E (mg α-TE)	1.1	1.1	1.1
Alpha-tocopherol (mg)	1.3	1.3	1.3

^1^ IF, infant milk formula; ^2^ scGOS/lcFOS (9:1), a specific prebiotic mixture consisting of short-chain galacto-oligosaccharides and long-chain fructo-oligosaccharides in a ratio of 9:1.

**Table 2:** Demographic characteristics of the per-protocol population for those subjects fully formula-fed ≤ 28 days of age.

	Statistic	Control w/o Prebiotics IF ^1^ (n = 28)	Control IF (n = 29)	Concept IF (n = 35)	Breastfed (n = 66)
**Infant characteristics**					
*Sex*					
Male	n (%)	**16 (57%)**	**16 (55%)**	**22 (63%)**	30 (45%)
Female	n (%)	12 (43%)	13 (45%)	13 (37%)	36 (55%)
*Ethnicity*					
Chinese	n (%)	**12 (43%)**	**11 (38%)**	**23 (66%)**	55 (83%)
Indian	n (%)	16 (57%)	13 (45%)	11 (31%)	8 (12%)
Malay	n (%)	0 (0%)	0 (0%)	0 (0%)	3 (5%)
Other	n (%)	0 (0%)	5 (17%)	1 (3%)	0 (0%)
*Birth characteristics*					
Weight (g)	Mean (SD)	**3159 (345)**	**3102 (319)**	**3059 (350)**	3138 (310)
Length(cm)	Mean (SD)	49.3 (1.8)	48.5 (1.5)	48.2 (1.6)	49.6 (2.1)
Head circumference (cm)	Mean (SD)	33.8 (0.9)	33.5 (1.2)	33.6 (1.4)	33.6 (1.0)
Caesarean section	n (%)	8 (29%)	4 (14%)	11 (31%)	10 (15%)
Gestational age (wk)	Mean (SD)	38.9 (1.1)	39.0 (1.1)	38.9 (0.9)	39.0 (1.1)
**Maternal characteristics**					
Maternal age (y)	Mean (SD)	29.1 (6.2)	29.7 (6.4)	29.6 (5.6)	30.5 (3.8)
Maternal university education (yes)	n (%)	2 (7%)	4 (14%)	4 (11%)	48 (73%)
Weight status ^2^					
Underweight	n (%)	6 (21%)	4 (14%)	5 (14%)	9 (14%)
Normal	n (%)	6 (21%)	14 (48%)	14 (40%)	38 (58%)
Overweight	n (%)	5 (18%)	7 (24%)	8 (23%)	17 (26%)
Obese	n (%)	11 (39%)	4 (14%)	8 (23%)	2 (3%)
Smoking status					
Before pregnancy (no)	n (%)	22 (79%)	18 (62%)	23 (66%)	64 (97%)
During pregnancy (no)	n (%)	25 (89%)	23 (79%)	26 (77%)	65 (99%)

^1^ IF, infant milk formula. ^2^ Weight status was based on pre-pregnancy BMI: underweight BMI <18.5 kg/m^2^, normal weight BMI 18.5 kg/m^2^ to <23 kg/m^2^, overweight BMI 23 kg/m^2^ to <27.5 kg/m^2^, obese BMI ≥ 27.5 kg/m^2^. Apparent differences between groups indicated in bold.

**Table 3:** Stool characteristics of the AST population of infants on study product or being fully breastfed ^1^.

Parameter	Statistic	Age	Control w/o Prebiotics IF ^2^	Control IF	Concept IF	Breastfed ^3^
Stool frequency (n/d)	Median (Q1–Q3; N)	1 mo	2.2 (1.1–3.7; 136) ^a^	2.6 (1.1–4.7; 115) ^a^	2.6 (1.4–3.9; 126) ^a^	4.9 (3.0–6.3; 114) ^b^
		2 mo	1.1 (0.7–2.1; 122) ^a^	1.1 (0.6–2.2; 104) ^a^	1.3 (0.7–2.3; 119) ^a^	2.6 (1.4–5.1; 17) ^b^
		3 mo	0.9 (0.4–1.7; 147) ^a^	1.0 (0.6–1.6; 132) ^a^	1.0 (0.6–1.8; 140) ^a^	1.7 (0.6–3.3; 75) ^b^
		4 mo	0.9 (0.4–1.4; 147) ^a^	1.0 (0.7–1.4; 134) ^b^	1.0 (0.6–1.7; 143) ^b^	1.3 (0.6–2.4; 67) ^c^
Stool consistency score	Mean (SD; N)	1 mo	2.2 (0.5; 136) ^a^	2.1 (0.5; 115) ^a^	2.0 (0.3; 126) ^b^	1.9 (0.3; 112) ^b^
		2 mo	2.1 (0.5; 122)	2.0 (0.4; 102)	2.0 (0.4; 118)	1.9 (0.2; 17)
		3 mo	2.1 (0.5; 145) ^a^	2.0 (0.4; 131) ^ab^	1.9 (0.5; 139) ^bc^	1.8 (0.4; 75) ^c^
		4 mo	2.1 (0.4; 144) ^a^	2.0 (0.4; 132) ^a^	1.9 (0.5; 142) ^b^	1.9 (0.4; 67) ^b^
Percentage of days with ≥1 watery stool	Mean (SD; N), %	1 mo	5.9 (17.6; 136) ^a^	9.3 (21.8; 115) ^ab^	10.3 (23.4; 126) ^b^	14.9 (30.8; 114) ^ab^
		2 mo	10.9 (26.4; 122)	9.3 (23.2; 104)	10.2 (22.3; 119)	8.4 (18.3; 17)
		3 mo	7.0 (20.7; 147) ^a^	8.8 (20.9; 132) ^ab^	13.5 (25.0; 140) ^bc^	15.1 (24.3; 75) ^c^
		4 mo	4.8 (17.9; 147) ^a^	10.5 (23.8; 134) ^b^	17.4 (30.9; 143) ^b^	16.2 (29.3; 67)^bc^
Percentage of days with ≥1 hard stool or no stool	Median (Q1–Q3; N), %	1 mo	0.0 (0.0–28.6; 136) ^a^	0.0 (0.0–28.6; 115) ^a^	0.0 (0.0–14.3; 126) ^a^	0.0 (0.0–0.0; 114) ^b^
		2 mo	14.3 (0.0–42.9; 122)	14.3 (0.0–42.9; 104)	14.3 (0.0–42.9; 119)	0.0 (0.0–14.3; 17)
		3 mo	28.6 (0.0–57.1; 147)	28.6 (0.0–57.1; 132)	28.6 (0.0–57.1; 140)	14.3 (0.0–42.9; 75)
		4 mo	28.6 (0.0–51.7; 147) ^a^	14.3 (0.0–42.9; 134) ^ab^	28.6 (0.0–42.9; 143) ^ab^	0.0 (0.0–57.1; 67) ^b^

^1^ The timing of randomization was flexible and, to evaluate the intervention products most stringently, the diary data of randomized infants before their first formula consumption (when still being breastfed) were not considered in the statistical analysis comparing intervention groups. These data were allocated to the group of breastfed infants instead. ^2^ IF, infant milk formula. ^3^ The visit at 2 months of age was optional for breastfed infants, resulting in a limited number of diaries (*n* = 17). N, number of infants per visit. ^a,b,c^ Different letters indicate a statistically significant difference between study group arms at a certain timepoint (*p* < 0.05).

## Data Availability

The data presented in this study are available on request from the corresponding author.

## Supplementary Materials

The following supporting information can be downloaded at: https://www.mdpi.com/article/10.3390/nu14030634/s1, Table S1: Demographic characteristics of the intervention groups in the ITT population; Table S2: Mean (SD) daily formula intake per intervention group of the PP population fully formula-fed before 28 days of age (ml/kg/d); Table S3: Tolerance parameters in the formula intervention groups of the AST population and the breastfed reference group.
