# Supplementary material for: An Infant Formula with Large, Milk Phospholipid-Coated Lipid Droplets Supports Adequate Growth and Is Well-Tolerated in Healthy, Term Asian Infants: A Randomized, Controlled Double-Blind Clinical Trial
Source: Nutrients. 2022 Feb 1;14(3):634. doi: 10.3390/nu14030634 (PMC8838783; doi:10.3390/nu14030634)
Supplement: Supplementary file 1 [file nutrients-14-00634-s001.zip › nutrients-1543355-supplementary.pdf]

Supplementary Table S1. Demographic characteristics of the intervention groups[1] in the ITT population

	Statistic	Control w/o prebiotics IMF[2] (n = 155)	Control IMF (n = 146)	Concept IMF (n = 152)
**Infant characteristics**				
Sex				
Male	n (%)	81 (52%)	77 (53%)	80 (53%)
Female	n (%)	74 (48%)	69 (47%)	72 (47%)
Ethnicity				
Chinese	n (%)	92 (59%)	83 (57%)	98 (65%)
Malay	n (%)	50 (32%)	50 (34%)	44 (29%)
Indian	n (%)	9 (6%)	5 (3%)	5 (3%)
Other	n (%)	4 (3%)	8 (6%)	5 (3%)
Birth characteristics				
Weight (g)	Mean (SD)	3135 (348)	3174 (355)	3158 (361)
Length(cm)	Mean (SD)	48.9 (1.9)	49.0 (1.8)	49.3 (2.0)
Head circumference (cm)	Mean (SD)	33.6 (1.2)	33.8 (1.3)	33.6 (1.2)
Caesarean section	n (%)	44 (28%)	39 (27%)	33 (22%)
Gestational age (wk)	Mean (SD)	38.9 (1.0)	39.0 (1.0)	39.0 (1.0)
**Maternal characteristics**				
Maternal age (y)	Mean (SD)	30.8 (5.4)	30.0 (5.2)	30.0 (4.8)
Maternal university education (yes)	n (%)	41 (27%)	49 (35%)	40 (28%)
Weight status[2]				
Underweight	n (%)	19 (13%)	21 (15%)	20 (14%)
Normal	n (%)	69 (45%)	69 (50%)	73 (51%)
Overweight	n (%)	35 (23%)	26 (19%)	26 (18%)
Obese	n (%)	29 (19%)	23 (17%)	25 (17%)
Smoking status				
Before pregnancy (no)	n (%)	130 (86%)	113 (81%)	115 (80%)
During pregnancy (no)	n (%)	144 (95%)	129 (93%)	125 (87%)

[1] Infants randomized to intervention formula before 4 months of age; [2]IMF; infant milk formula; [2]Weight status was based on pre-pregnancy BMI: Underweight BMI <18.5 kg/m^2, Normal weight BMI 18.5 kg/m^2 to < 23 kg/m^2, Overweight BMI 23 kg/m^2 to < 27.5 kg/m^2, Obese BMI ≥ 27.5 kg/m^2 .

Supplementary Table S2. Mean (SD) daily formula intake per intervention group of the PP population fully formula fed before 28 days of age (ml/kg/d)

**Age (months)**	Control w/o prebiotics IMF[1] (n = 28)	Control IMF (n = 29)	Concept IMF (n = 35)
1	174 (44)	169 (42)	183 (38)
2	165 (25)	172 (42)	161 (34)
3	145 (28)	145 (38)	144 (30)
4	149 (26)	134 (34)	136 (29)

[1]IMF; infant milk formula

Supplementary Table S3. Tolerance parameters of the AST population of infants on study product or being fully breastfed

Parameter	Statistic	Age	Severity	Control w/o prebiotics IMF[2]	Control IMF	Concept IMF	Breastfed[3]
Diarrhea incidence[3]	n (%, N)	1 mo	--	6 (4.4%; 136)	6 (5.2%; 115)	7 (5.6%; 126)	13 (11.4%; 114)
	n (%, N)	2 mo	--	10 (8.2%; 122)	5 (4.8%; 104)	3 (2.5%; 119)	2 (11.8%; 17)
	n (%, N)	3 mo	--	7 (4.8%; 147)	4 (3.0%; 132)	8 (5.7%; 140)	7 (9.3%; 75)
	n (%, N)	4 mo	--	3 (2.0%; 147)**	9 (6.7%; 134)	10 (7.0%; 143)	4 (6%; 67)
Regurgitation[4]	n (%)	1 mo*	Absent	63 (46%)	52 (45%)	66 (52%)	37 (33%)
			Mild	56 (41%)	45 (39%)	49 (39%)	68 (60%)
			Moderate	17 (13%)	17 (15%)	10 (8%)	9 (8%)
			Severe	0 (0%)	1 (1%)	1 (1%)	0 (0%)
		2 mo#	Absent	56 (46%)	48 (46%)	63 (53%)	4 (24%)
			Mild	59 (48%)	44 (42%)	40 (34%)	11 (65%)
			Moderate	7 (6%)	12 (12%)	15 (13%)	2 (12%)
			Severe	0 (0%)	0 (0%)	1 (1%)	0 (0%)
		3 mo§	Absent	85 (58%)	75 (57%)	95 (68%)	34 (45%)
			Mild	53 (36%)	46 (35%)	40 (29%)	36 (48%)
			Moderate	24 (6%)	11 (8%)	4 (3%)	5 (7%)
			Severe	1 (0%)	0 (0%)	1 (1%)	0 (0%)
		4 mo§	Absent	97 (66%)	86 (64%)	101 (71%)	35 (52%)
			Mild	46 (31%)	43 (32%)	37 (26%)	30 (45%)
			Moderate	4 (3%)	4 (3%)	4 (3%)	2 (3%)
			Severe	0 (0%)	1 (1%)	1 (1%)	0 (0%)

[1] The timing of randomisation was flexible and to evaluate the intervention products most stringently, the diary data of randomized infants before their first formula consumption (when still being breastfed) was not considered in the statistical analysis comparing intervention groups. This data was allocated to the group of breastfed infants instead. [2]IMF, infant milk formula. [3]The visit at 2 months of age was optional for breastfed infants, resulting in a limited number of diaries (n=17). [3]Applying the WHO definition of having ≥ 3 watery stools on 1 day; incidences were tested using the MN test. [4]Based on worst score reported during 7-d period prior to visit, total number of infants per visit similar as reported for the diarrhea incidence. *BF reference significantly different (distribution) compared to formula intervention groups ($P < 0.05$). **Statistically significant difference between Control w/o prebiotic and Concept IMF group

($P < 0.05$; MN approach). [#]Statistically significant different distribution between Control w/o prebiotic and Concept IMF group ($P = 0.032$). [§] BF reference significantly different (distribution) compared to Concept IMF intervention group ($P < 0.05$).
